# Neighbours' Breeding Success and the Sex Ratio of Their Offspring Affect the Mate Preferences of Female Zebra Finches

**DOI:** 10.1371/journal.pone.0029737

**Published:** 2011-12-28

**Authors:** Dominique Drullion, Frédérique Dubois

**Affiliations:** Département de sciences biologiques, Université de Montréal, Montréal, Quebec, Canada; Monash University, Australia

## Abstract

Several hypotheses on divorce predict that monogamous pairs should split up more frequently after a breeding failure. Yet, deviations from the expected pattern “success-stay, failure-leave” have been reported in several species. One possible explanation for these deviations would be that individuals do not use only their own breeding performance (i.e., private information) but also that of others (i.e., public information) to decide whether or not to divorce. To test this hypothesis, we investigated the relative importance of private and public information for mate choice decisions in female zebra finches (*Taeniopygia guttata*).We manipulated the reproductive performance of breeding pairs and measured females' preferences for their mate and the neighbouring male first following pair formation and then seven weeks later when all females had laid eggs and the young were independent. Although all females reduced their preference for their mate after a breeding failure, the decrease was significant only when the neighbouring pair had reproduced successfully. Furthermore, there was no evidence that females biased the sex ratio of their offspring according to their mate's attractiveness. On the other hand, after reproduction, both successful and unsuccessful females increased their preferences for males who had produced a larger proportion of sons. Despite the fact that other mechanisms may have also contributed to our findings, we suggest that females changed their mate preferences based on the proportion of sons produced by successful males, because offspring sex ratio reflects the male's testosterone level at the moment of fertilization and hence is an indicator of his immune condition.

## Introduction

Mate choice may incur important costs, such as time and energy spent in searching for and assessing potential partners [1]. Such costs may frequently force individuals prospecting for a mate to make a sub-optimal choice. This is particularly likely to happen when conditions required for reproduction last for only a short time, because these constraints prevent individuals from sampling a large number of potential partners [2]. Also, in monogamous species, females that breed late in the season have an increased risk of reproducing with a low-quality male, because males become unavailable for other females following pair formation. Thus, the cost of choosiness increases over the course of the breeding season as new pairs are formed. Because individuals who made an incorrect choice reproduce poorly, they have a high potential to subsequently improve their mating status with a new mate. Hence, several authors have suggested that monogamous birds should use their breeding performance to decide whether or not to divorce and have predicted that mate switching should occur more frequently after a breeding failure (Incompatibility hypothesis [3]; Better option hypothesis [4]; see review [5]). Accordingly, results from a meta-analysis based on 35 monogamous species revealed a significant relationship between mate switching and breeding success [6]. However, the overall effect of this relationship was rather weak as breeding success seems to have only a minor effect on the probability of divorce in several species [6], [7]. In fact, several experiments have even shown that divorces occurred more frequently among successful pairs [8], [9]. One possible explanation for these deviations from the expected pattern “success-stay, failure-leave” would be that individuals do not use only their own breeding success (i.e. private information), but also that of conspecifics (i.e. public information) to make a decision [10], [11]. Indeed, divorce is adaptive for at least one of the pair members if it manages to improve its breeding success by obtaining a more suitable partner. The probability of doing so, however, depends not only on the breeding success of the pair, but also on the availability and quality of potential partners within the population. Individuals would therefore benefit from observing the reproductive success of others to gauge their own relative breeding performance and the quality of new potential mates. Individuals could then use the cues provided by conspecifics either to find a new mate directly [4] or to simply assess the probability of improving their own breeding situation [5]. In this case, only pairs whose breeding performance is below the population mean would split, while those above the population mean would be maintained, regardless of the absolute reproductive success of the pair. There is some evidence that individuals are influenced by the reproductive success of others when they choose a breeding site [12]–[14]. For instance, Doligez et al. [15] found that the immigration rate of Collared flycatchers (*Ficedula albicollis*) was higher when the local breeding success of the site was experimentally increased, while the proportion of emigrants increased when local offspring quantity or quality decreased. Both departure and settlement decisions, however, varied among individuals depending on their previous breeding experience and hence were also affected by private information [15].

In the present study, we investigated the relative importance of private and public information for mate-choice decisions in a monogamous species. We manipulated the breeding success of zebra finch pairs and measured females' preference for their mate or the neighbouring male at two different moments: shortly after pair formation and seven weeks later when all the females had laid eggs and the young produced by successful pairs were independent. Because all zebra finch pairs reproduced simultaneously, females could use both their own breeding performance and that of the neighbouring pair to form a mate preference. If both private and public information are important, we predict that females should reduce their preference for their mate after a breeding failure. However, the change in females' preferences should be more pronounced among unsuccessful females whose neighbouring pairs reproduced successfully. Furthermore, as females are predicted to adjust the sex ratio of their offspring according to their mate's attractiveness [16], we would expect that the change in unsuccessful females' preferences will be affected not only by the breeding performance of the neighbouring pair but also by the sex ratio of their offspring. More precisely, we predict that successful females should produce a larger proportion of sons when mated with an attractive male and that unsuccessful females consequently should prefer neighbouring males whose sex ratio is more male-biased. Indeed, sex allocation theory predicts that females paired to more attractive males should bias their offspring sex ratio in favour of sons. Supporting this expectation, Burley [17] found that zebra finch females mated with attractive red-banded males produce more sons as compared to females mated with unattractive green-banded males [17]. This is so because the variance in reproductive success is greater in males than in females and more attractive males therefore achieve greater reproductive success than less attractive ones [17]. Thus, if attractiveness reflects male genetic quality [18], [19], sons inherit the quality of their father and females mated with attractive males would benefit from producing attractive sons.

## Methods

### Ethics statement

The experiments described in this study were approved by the Animal Care Committee of the Université de Montréal (animal care permit #06-187) and conformed to all guidelines of the Canadian Council on Animal Care.

### Subjects

We used 36 (18 males and 18 females) commercially purchased adult zebra finches [20] that had never been in contact with each other before the experiment. Females were paired randomly with males and each pair was then kept for all the duration of the experiment in an individual cage (38×38×48 cm) with a 14∶10 hour light:dark photoperiod at approximately 23°C. Birds had *ad libitum* access to seeds, water, cuttlefish bone and nesting material. In addition, we supplemented their diet each day with an egg yolk rearing mixture. All the pairs were kept in the same housing room, and hence they could hear each other. However, we placed an opaque partition on one side of each cage, and arranged the cages so that each pair could see only one other pair (see details below).

### Apparatus and experimental procedure

**Figure 1:**
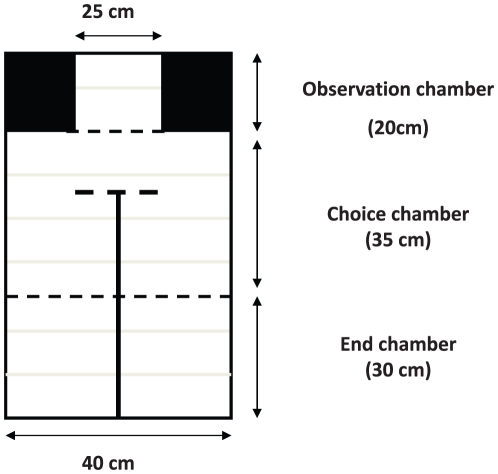
Plan view of the mate choice apparatus. The grey lines represent the perches while the black lines correspond to the partitions that are either opaque (full lines) or clear (dashed lines).

We measured females' preferences in a mate choice apparatus that consisted of three chambers (Figure 1): an observation chamber (20×25×20cm) that housed the female before she was released into the central choice chamber (35×40×35cm), and an end chamber (30×40×35cm) that was divided into two symmetrical compartments, each housing a single male. The chambers were covered and separated by Plexiglas partitions for behavioural observations and videotaping. We tested each female four times: twice only five days after pair formation when the pairs had not reproduced yet, and two more times seven weeks later when all the females had laid eggs and the young produced by successful pairs were nutritionally independent and could be sexed by appearance. Twenty-four hours before each preference test, females were separated from their breeding partner. In addition, each female was placed in the mate choice apparatus two hours per day during the two days preceding the formation of the pairs to allow her to become familiar with the test environment.

**Figure 2:**
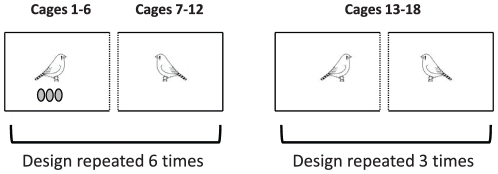
Configuration of the cages in the housing room. Among the 18 pairs, 6 reproduced successfully (cages 1 to 6) whereas the 12 other pairs reproduced unsuccessfully (cages 7 to 18). The cages were arranged so that each pair could observe only one other pair through a transparent partition (dashed lines).

We randomly designated six of the 18 zebra finch pairs as “successful pairs” (S), while the others were designated as “unsuccessful pairs” (U). Unsuccessful pairs reproduced normally, as successful pairs, but when unsuccessful females started to incubate, we replaced fertile eggs by sterile eggs that had been previously collected in unisex female cages. Every day, we verified whether females had laid new eggs and, if so, we replaced them. Because we did not change clutch sizes, it is unlikely that the parental behaviour of the pair members was affected by this procedure. To control for the effect of manipulation, we also removed all the eggs laid by successful females, but put the same eggs back in their respective nests after a few seconds. The cages were arranged so that the six successful pairs could each observe one unsuccessful pair, while the six remaining unsuccessful pairs could each observe one other unsuccessful pair (Figure 2). Thus, we get three different types of females: 6 females reproduced successfully but the neighbouring pair reproduced unsuccessfully (cages 1 to 6); 6 females reproduced unsuccessfully but the neighbouring pair reproduced successfully (cages 7 to 12); finally 6 females reproduced unsuccessfully and the neighbouring pair too (cages 13 to 18).

### Measurement of mate preferences

We measured female preferences before and after reproduction. Each time, we tested each female twice in a 24 h interval. For each trial, females were placed in the observation chamber of the choice apparatus in front of two males, their breeding partner and the male of the neighbouring pair, each in one of the end chamber compartments. The position of the two males was switched between two consecutive trials to control for female side bias in the choice chamber. After an acclimatization period, during which males were separated from the female by an opaque partition, and an observation period of 20 min each, we measured the time spent by females in front of each male for 20 min. To measure females' preferences, we considered only the time they spent on the two perches in front of each male, while the time they spent on the back perch or on the ground was excluded from analyses (Figure 1). Note, however, that no female has spent more than 10% of time in the neutral zone.

### Statistical analyses

We calculated female preference as the time spent by females in front of their preferred male divided by the total of time spent in front of the two ends of the choice chamber. We used paired t-tests to examine whether females changed their preference between trials, as well as to test if successful and unsuccessful females whose cages were side by side differed in their preferences, both before and after reproduction. We also compared the preferences of successful and unsuccessful females from neighbouring pair cages with one-sample t-tests and performed Spearman rank correlations between fledgling sex ratio and females' preferences. All statistical analyses were conducted with SPSS 16.0 for PC.

## Results

### Changes in female preferences in relation to breeding performance

**Figure 3:**
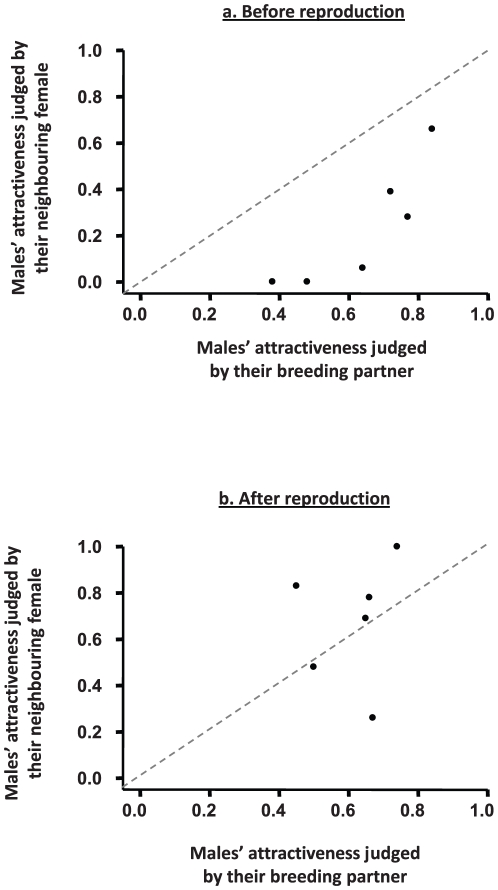
Percent of choosing time spent with each of the 6 successful males by their breeding partner (*x* axis) or the neighbouring female (*y* axis) before (a) and after (b) reproduction.

**Figure 4:**
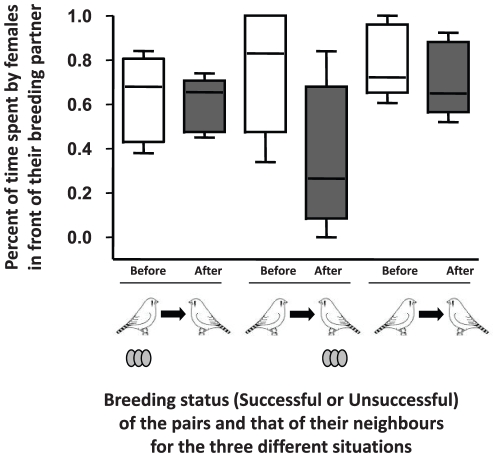
Mean preference of females for their breeding partner versus the neighbouring male, for the three different configurations: 1) successful females whose neighbouring pair reproduced unsuccessfully (*N* = 6), 2) unsuccessful females whose neighbouring pair reproduced successfully (*N* = 6) and 3) unsuccessful females whose neighbouring pair reproduced unsuccessfully (*N* = 6). The white and grey boxes represent the percent of time (±SEM) spent by females in front of their breeding partner before and after reproduction, respectively.

All females but two preferred their own breeding partner over the neighbouring male before reproduction. As a consequence the mean percent of time spent by females in front of their breeding partner before mating was significantly higher than 50% (72.9±4.8%; unilateral t-test: t_17_ = 4.239, *P*<0.001), and although females in successful pairs spent less choosing time in front of their breeding partner compared to those in unsuccessful pairs, the difference was not significant (mean percent of time successful females spent with their breeding partner (X ± SE): successful females : 63.9±7.2%, unsuccessful females: 77.3±5.9%, t-test: t_16_ = −1.370, *P* = 0.190). In addition, because almost all females strongly preferred their own partner over their neighbour, the percent of time that females spent in front of the successful males was significantly higher when the male was the breeding partner rather than the neighbour (paired t-test: t_5_ = 7.032, *P* = 0.001; Figure 3a). After reproduction, females' mate preferences were affected both by their own breeding success and that of the neighbouring pair (Figure 4). More precisely, we found that successfully reproducing females maintained their initial preference for their breeding partner (paired t-test: t_5_ = 0.313, *P* = 0.767). On the contrary, unsuccessful females significantly decreased the relative time spent with their breeding partner after reproductive failure (paired t-test: t_11_ = 2.290, *P* = 0.043). The magnitude of the decrease, however, was different depending on whether the neighbouring pair had reproduced successfully or not: unsuccessful females that had observed another unsuccessful pair decreased their preference for their breeding partner by only 8.0%, which is not significantly different from zero (unilateral t-test: t_5_ = 0.134, *P* = 0.134), whereas, unsuccessful females whose neighbours had reproduced successfully spent 42.5% more time with the neighbouring male after reproducing, which is a marginally significant change (unilateral t-test: t_5_ = 2.284, *P* = 0.0534). Finally, after reproduction, the successful males were preferred over their unsuccessful neighbours by both their breeding partner and the neighbouring female. As a consequence, the percent of time that females spent in front of the successful males was not significantly different when the male was the breeding partner or the neighbour (paired t-test: t_5_ = 0.553, *P* = 0.604; Figure 3b).

### Variation in fledgling sex-ratio

Because we did not determine the sex of the embryos in the eggs, we knew only the sex for the 21 birds that have reached the fledgling stage. The successful females all produced between 2 and 5 offspring with a proportion of males that varied considerably among females from 0% to 67%. The percent of choosing time spent by successful females with their breeding partner before reproduction negatively correlated with the proportion of males in the brood (Spearman rank correlation coefficient = −0.698, *N* = 6, *P* = 0.123), although the relation was not significant. On the other hand, the mean change in females' preferences was significantly positively correlated with the sex ratio of their offspring (Spearman rank correlation coefficient = 0.820, *N* = 6, *P* = 0.046), indicating that females that produced more daughters decreased the most their preference for their breeding partner. Finally, the fledgling sex ratio of the successful pairs also affected the mating preference of the neighbouring unsuccessful females. More precisely, the change in females' preferences was positively correlated to the sex ratio of the neighbour's pair (Spearman rank correlation coefficient  = 0.820, *N* = 6, *P* = 0.046). Thus, unsuccessful females spent more time with the neighbouring males when the successful neighbours had produced a larger proportion of sons.

## Discussion

Although almost all females strongly preferred their breeding partner over the neighbouring male before reproduction, only those that had reproduced successfully maintained their initial preference after mating. On the other hand, unsuccessful females increased their preference for the neighbouring male, but the increase was significant only when the neighbouring pair had reproduced successfully. Despite our conclusions being based on a laboratory experiment in which the cages were arranged to facilitate direct comparison of breeding performances between neighbouring females, we feel confident that this experimental setup mimics natural conditions. Indeed, zebra finches live in very large colonies in which most breeding pairs reproduce synchronously [21]. Females, therefore, are likely to have great opportunities to observe their neighbours' reproductive performances and hence to assess their chances of improving their breeding situation. If the percent of time spent by females with each male is a good indicator of their willingness to divorce, then our study demonstrates that monogamous zebra finch females use both their own breeding success and that of the neighbouring pair to decide whether or not to divorce, thereby providing supplementary evidence that public information use is not restricted to polygynous species, but is important in monogamous species as well [20], [22]–[23]. Furthermore, given that only females who could expect to gain a better quality male lost their initial preference for their partner, our results strongly suggest that divorce in zebra finches might be better explained by the better option hypothesis [4] than the incompatibility hypothesis [3]. Indeed, the incompatibility hypothesis states that divorce should be a mutual decision by pair members [3]. On the contrary, the better option hypothesis predicts that divorce should be initiated by one partner which leaves the other of a victim as its choice when it has an opportunity to improve its breeding status [4]. This hypothesis therefore requires that at least one pair-member has already found a better option to initiate a divorce, and then is more appropriate to explain our results. Finally, as expected, the change in females' preferences was affected not only by the breeding performance of their neighbours relative to their own, but also by the sex ratio of their offspring. More precisely, we found that the magnitude of the change in unsuccessful females' mate preferences was positively correlated to the proportion of sons produced by the neighbouring pair. This result is consistent with our prediction and apparently supports the hypothesis that females mated to attractive males tend to bias their brood sex ratio towards sons [17]. Contradicting this hypothesis, however, there was little effect of females' preference on their offspring sex ratio, with even a tendency for females exhibiting the strongest initial preferences for their breeding partner to produce more daughters. Thus, other mechanisms would better account for our results. In particular, although our findings have to be interpreted cautiously given our small sample size, they suggest that females would use the proportion of sons produced by males to assess their quality. Indeed, we found no evidence that females adjusted the sex ratio of their offspring according to their level of attachment to their partner. After reproduction, however, both successful and unsuccessful females changed their mate preferences based on the proportion of sons produced by successful males, probably because offspring sex ratio reflects the male's testosterone level at the moment of fertilization and hence may be used as an indicator of quality of his immune system. There is support for this explanation. First, although a number of studies have reported positive correlations between male attractiveness and brood sex ratios [17], [24]–[27], Rutstein et al. [28] reported that mate attractiveness did not affect yolk androgen investment, and then also had no effect on the primary sex ratio of female zebra finch broods. Second, the role of maternal testosterone in the determination of the primary sex ratio of zebra finch offspring has already been demonstrated [29]. It is therefore very likely that the sex ratio of the offspring is affected by the testosterone levels of both parents around the time of conception [30]. Third, given that testosterone is immunosuppressive, we expect males with high androgen levels to have better genetic resistance to parasites and disease [31]. For these reasons, females should prefer males with high testosterone levels and hence those that produce male-biased sex ratios. Accordingly, we found that all females, regardless of their own breeding success, increased their preference for the males that had produced a larger proportion of sons. Thus, despite the relative level of attractiveness of the males in the successful pairs initially differing significantly among females, the difference disappeared after reproduction.

In conclusion, our study indicates that zebra finch females use both their breeding performance and that of their conspecifics to assess the relative quality of their mate and modify their preference accordingly. Our results therefore provide a simple explanation for why deviations from the expected pattern “success-stay, failure-leave” frequently occur. Furthermore, although other factors, such as the female's body condition [32] or the maternal corticosterone level [33]–[35] may have contributed to the observed variation in offspring sex ratio, our results suggest that the proportion of sons in broods would be affected by the level of male testosterone as well, and hence might be used by females to assess the immune condition of potential partners. Our conclusions, however, must be viewed carefully and would require confirmation with studies using larger samples and measuring males' testosterone concentration.
